# Clinical and genetic profile of lower limb dystonia: a dual-center retrospective study in China

**DOI:** 10.3389/fneur.2026.1957431

**Published:** 2026-10-30

**Authors:** Jun Ma, Shanglin Li, Lin Wang, Yingmai Yang, Wenjing Jiang, Xinhua Wan, Yiming Liu

**Affiliations:** 1 Department of Geriatric Medicine and Laboratory of Gerontology and Anti-Aging Research, Qilu Hospital of Shandong University, Jinan, China; 2 Department of Neurology, The First Hospital of Tsinghua University, Beijing, China; 3 Department of Neurology, Peking Union Medical College Hospital, Chinese Academy of Medical Sciences, Beijing, China; 4 Department of Neurology, Qilu Hospital of Shandong University, Jinan, China

**Keywords:** China, dystonia, gene, lower limb, phenotype

## Abstract

**Background:**

Lower limb dystonia (LLD) is a clinically significant form of dystonia that can substantially impair gait and quality of life. Its clinical presentation is highly heterogeneous, and investigations into underlying genetic causes remain limited. This study aimed to characterize the clinical and genetic profile of LLD in a Chinese cohort and to explore potential genotype–phenotype correlations.

**Methods:**

Patients diagnosed with LLD without an acquired cause were retrospectively enrolled at two movement disorder centers in China between January 2016 and December 2024. Clinical characteristics were collected, and genetic testing results were comprehensively analyzed.

**Results:**

A total of 68 patients with LLD without an acquired cause were included. The median age at dystonia onset was 14.0 years (IQR, 6.3–23.0 years), with 46 patients (67.6%) developing symptoms before the age of 20 years. Foot dystonia was the most common initial manifestation, presenting as foot inversion (25.0%) and foot plantarflexion (20.6%). At the last follow-up, 35 patients (51.5%) remained restricted to lower limbs involvement (LLD group), whereas the remaining 33 patients (48.5%) developed dystonia spreading to other anatomical regions (LLD-plus group). Pathogenic or likely pathogenic variants were identified in 29 patients (42.6%), involving *TOR1A* (*n* = 8), *GCH1* (*n* = 6), *KMT2B* (*n* = 5), *PRKN* (*n* = 5), *TH* (*n* = 2), *SGCE* (*n* = 1), *PINK1* (*n* = 1), and *PANK2* (*n* = 1). The distribution of causative genes differed between the two groups. *GCH1* and *PRKN* were the most frequently implicated genes in the LLD group, whereas *TOR1A* and *KMT2B* were the predominant causative genes in the LLD-plus group.

**Conclusion:**

LLD in the Chinese cohort is a clinically and genetically heterogeneous disorder, with a substantial proportion of patients harboring identifiable monogenic causes.

## 1 Introduction

Dystonia is a movement disorder characterized by sustained or intermittent abnormal movements, postures, or both (1, 2). Its clinical presentation is highly heterogeneous, encompassing a wide range of ages at onset (AAO), diverse body distributions, and various associated neurological features. Lower limb dystonia (LLD) represents a distinct and clinically significant phenotype that can substantially impair gait and quality of life. The prototypic manifestations include foot torsion, with inversion of the foot at the ankle joint, usually accompanied by flexion of the forefoot and toes (3). While LLD frequently manifests in childhood or adolescence with a high propensity for generalization, adult-onset cases are relatively rare and typically remain focal (4–6). The etiology of LLD is increasingly linked to genetic factors. Notably, in early-onset cases, a monogenic cause is frequently identifiable. Key causative genes include *TOR1A*, *GCH1*, *KMT2B*, and *PRKRA* (7). In contrast, the genetic basis of adult-onset LLD is less defined, though emerging evidence suggests that genetic factors may still play a pivotal role in its pathogenesis (8–10).

To date, only a few studies have reported the clinical features of LLD (3, 6, 11, 12), and systematic investigations into its underlying genetic cause remain limited, especially in Asian populations. In this study, we conducted a retrospective analysis to characterize the clinical features and explore the genetic basis of patients with LLD in China.

## 2 Methods

### 2.1 Participants

This retrospective study enrolled patients with LLD at Peking Union Medical College Hospital and Qilu Hospital of Shandong University between January 2016 and December 2024. LLD was defined as lower limb onset dystonia in which the initial dystonic manifestation involved one or both lower limbs. The diagnosis was established by two experienced movement disorder specialists (Dr. XH Wan and Dr. YM Liu). Patients who were suspected of acquired structural, drug-induced, or functional etiologies were excluded based on medical history, neurological examination, medication and toxin exposure history, and available laboratory and neuroimaging investigations.

Clinical characteristics of all enrolled patients were retrospectively collected, including demographic data, initial symptoms, family history, follow-up duration, and anatomical distribution of dystonia (focal, segmental, multifocal, hemidystonia, or generalized dystonia). Adult-onset dystonia was defined as dystonia onset after the age of 20 years, in accordance with established diagnostic criteria (1). Associated neurological features (e.g., parkinsonism, myoclonus, chorea, or cognitive impairment) were systematically recorded. Follow-up duration was defined as the interval between the initial diagnosis of lower limb dystonia and the date of the last clinical assessment or data collection. Patients with dystonia restricted to the lower limb(s) were classified into the LLD group, whereas those who initially presented with lower limb dystonia and subsequently developed involvement of other anatomical regions were classified into the LLD-plus group. According to the current classification criteria (2), patients in the LLD group were categorized into focal dystonia (unilateral lower limb involvement) or multifocal dystonia (bilateral lower limbs involvement).

This study was approved by the Ethics Committee of Qilu Hospital of Shandong University and Peking Union Medical College Hospital. Written informed consent was obtained from all participants or their legal guardians for clinical data collection and genetic analysis.

### 2.2 Genetic analysis

#### 2.2.1 Next-generation sequencing (NGS)

Genomic DNA was extracted from peripheral blood samples of all participants. Genetic testing was performed using either targeted next-generation sequencing with a 148-gene panel associated with movement disorders in 27 patients enrolled between January 2016 and December 2016, or whole exome sequencing (WES) in 41 patients between January 2017 and December 2024. The complete list of genes included in the 148-gene targeted NGS panel was provided in Supplementary Table 1. The detailed methods for targeted NGS and WES were described in Supplementary materials. All identified variants with potential clinical significance were interpreted according to the American College of Medical Genetics and Genomics (ACMG) guidelines (13). Segregation analysis was performed whenever DNA samples from available family members could be obtained. Variants classified as pathogenic or likely pathogenic were considered molecularly diagnostic.

#### 2.2.2 Multiplex ligation-dependent probe amplification (MLPA)

In this cohort, 6 patients underwent additional screening for copy number variants (CNVs) in *DJ1*, *ATP13A2*, *PINK1*, *PRKN*, *UCHL1*, *SNCA*, *LRRK2*, and *GCH1* using multiplex ligation-dependent probe amplification (MLPA). Of these six patients, five had previously undergone targeted NGS using the 148-gene panel, and one had previously undergone WES. MLPA analysis was performed using commercially available kits (MRC-Holland, Amsterdam, The Netherlands), and data were analyzed using Coffalyser software.^1^

### 2.3 Statistical analysis

Statistical analyses were performed using SPSS version 26.0 (IBM Corp.). Continuous variables were summarized as mean ± standard deviation (SD) or median with interquartile range (IQR), and categorical variables were presented as frequencies and percentages. Comparisons of continuous variables were conducted using the Student’s *t*-test or Mann–Whitney *U* test, as appropriate. Categorical variables were compared using the chi-square test or Fisher’s exact test. A two-sided *p*-value <0.05 was considered statistically significant.

## 3 Results

### 3.1 Demographic and clinical characteristics

The demographic and clinical characteristics of the cohort were summarized in Table 1. A total of 68 patients with lower limb dystonia (33 males, 35 females) were enrolled in this study, including 42 patients from Peking Union Medical College Hospital and 26 from Qilu Hospital of Shandong University. The median age at dystonia onset was 14.0 years (IQR, 6.3–23.0 years), and 46 patients (67.6%) developed symptoms before the age of 20 years. Among the cohort, 55 patients (80.9%) presented with isolated dystonia, and the remaining 13 patients had combined/complex dystonia. The associated features included myoclonus (*n* = 1), parkinsonism/tremor (*n* = 8), dysarthria (*n* = 2), and cognitive impairment (*n* = 3). A positive family history was reported in 12 patients (17.6%). The most common initial manifestation was foot dystonia, including foot inversion (*n* = 17, 25.0%) and foot plantarflexion (*n* = 14, 20.6%).

**Table 1:** Demographic and clinical characteristics of patients with lower limb dystonia.

Variable	Total (*n* = 68)	LLD group (*n* = 35)	LLD-plus group (*n* = 33)	*p*-value^a^
Sex, *n* (%)				0.225
Males	33 (48.5)	14 (40.0)	19 (57.6)	
Females	35 (51.5)	21 (60.0)	14 (42.4)	
Age at onset, y (median, IQR)	14.0 (6.3–23.0)	21.0 (12.0–34.0)	8.0 (4.5–15.0)	**<0.001**
Disease duration, y (median, IQR)	12.7 (9.2–19.7)	10.9 (9.0–15.3)	15.0 (10.0–24.9)	**0.025**
Follow-up duration, y (median, IQR)	4.8 (3.1–5.6)	5.2 (3.2–5.7)	4.7 (3.0–5.4)	0.531
Positive Family history, *n* (%)	12 (17.6)	5 (14.3)	7 (21.2)	0.454
Age at onset distribution, *n* (%)				**0.002**
Infancy (≤2 years)	3 (4.4)	0 (0.0)	3 (9.1)	
Childhood (>2–12 years)	29 (42.6)	10 (28.6)	19 (57.6)	
Adolescence (>12–20 years)	14 (20.6)	7 (20.0)	7 (21.2)	
Early adulthood (>20–40 years)	18 (26.5)	14 (40.0)	4 (12.1)	
Late adulthood (>40 years)	4 (5.9)	4 (11.4)	0 (0.0)	
Dystonia distribution, *n* (%)				**<0.001**
Focal	16 (23.5)	16 (45.7)	0 (0.0)	
Segmental	9 (13.2)	0 (0.0)	9 (27.3)	
Multifocal	24 (35.3)	19 (54.3)	5 (15.2)	
Generalized	19 (27.9)	0 (0.0)	19 (57.6)	
Associated features, *n* (%)				0.154
Isolated dystonia	55 (80.9)	26 (74.3)	29 (87.9)	
Combined/complex dystonia	13 (19.1)	9 (25.7)	4 (12.1)	
With myoclonus	1 (1.5)	0 (0.0)	1 (3.0)	
With parkinsonism/tremor	8 (11.8)	7 (20.0)	1 (3.0)	
With dysarthria	2 (2.9)	1 (2.9)	1 (3.0)	
With cognitive impairment	3 (4.4)	2 (5.7)	1 (3.0)	
Initial symptom, *n* (%)				
Foot inversion	17 (25.0)	11 (31.4)	6 (18.2)	0.207
Foot eversion	1 (1.5)	0 (0.0)	1 (3.0)	0.485
Foot plantarflexion	14 (20.6)	5 (14.3)	9 (27.3)	0.186
Toe flexion	5 (7.4)	3 (8.6)	2 (6.1)	1.000
Toe extension (including striatal toe)	2 (2.9)	1 (2.9)	1 (3.0)	1.000
Leg extension/ flexion/external rotation	12 (17.6)	6 (17.1)	6 (18.2)	0.911
Unclassed LLD	21 (30.9)	13 (37.1)	8 (24.2)	0.250
With P/LP variants, *n* (%)	29 (42.6)	12 (34.3)	17 (51.5)	0.151

a Significant difference was indicated in bold.

LLD, lower limb dystonia; IQR, interquartile range; P, pathogenic; LP, likely pathogenic.

The median follow-up duration of the cohort was 4.8 years (IQR, 3.1–5.6 years). Based on clinical assessment at the last follow-up, 35 patients (51.5%) were classified into the LLD group, whereas 33 patients (48.5%) were classified into the LLD-plus group. The follow-up duration was comparable between the two groups [median (IQR): 5.2 (3.2–5.7) vs. 4.7 (3.0–5.4) years; *p* = 0.531]. Within the LLD-plus group, 19 patients (57.6%) presented with generalized dystonia, 5 (15.2%) had multifocal dystonia, and the remaining 9 (27.3%) exhibited segmental dystonia. Patients in the LLD-plus group had a significantly younger age at dystonia onset compared with those in the LLD group [median (IQR): 8.0 (4.5–15.0) vs. 21.0 (12.0–34.0) years; *p* < 0.001].

### 3.2 Genetic findings

In this cohort, 29 patients (42.6%) were identified to carry pathogenic or likely pathogenic (P/LP) variants in genes associated with dystonia and other movement disorders, including 12 patients in the LLD group and 17 patients in the LLD-plus group. These variants were summarized in Figure 1 and detailed in Tables 2, 3, Supplementary Tables S2, S3. The implicated genes were *TOR1A* (*n* = 8), *GCH1* (*n* = 6), *KMT2B* (*n* = 5), *PRKN* (*n* = 5), *TH* (*n* = 2), *SGCE* (*n* = 1), *PINK1* (*n* = 1), and *PANK2* (*n* = 1). Among these patients, the majority (23/29, 79.3%) had early-onset symptoms (before the age of 20 years), while the remaining six patients (20.7%) were classified as adult-onset. Additionally, five patients (7.4%) were found to carry variants of uncertain significance (VUS), including *PLA2G6* (*n* = 2), *PRKN* (*n* = 1), *ANO3* (*n* = 1) and *KMT2B* (*n* = 1) (Supplementary Tables S4, S5). No clinically relevant genetic variants were detected in the remaining 34 patients (50.0%).

**Figure 1:**
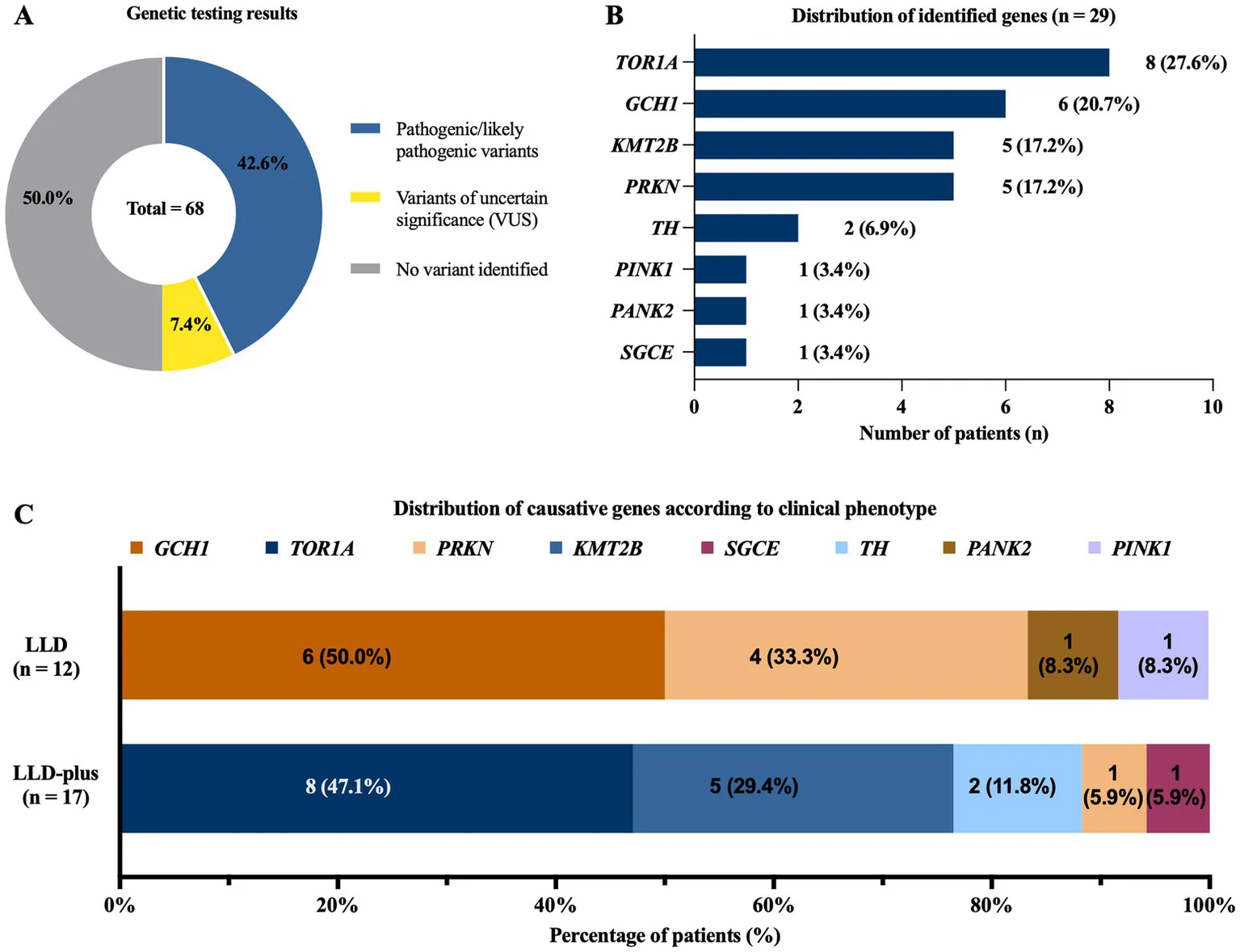
Genetic analysis results of the present cohort. **(A)** Overview of genetic testing findings; **(B)** Distribution of pathogenic or likely pathogenic variants in causative genes. **(C)** Distribution of pathogenic or likely pathogenic variants across clinical subgroups (LLD group and LLD-plus group) based on dystonia distribution at the last follow-up.

**Table 2:** Overview of identified pathogenic and likely pathogenic variants in the LLD group.

No.	AAO/sex	Initial LLD symptom	Dystonia type	Other associated disorders	Family history	Gene	cDNA	Protein	Variant type	Het/hom	Inherited	ACMG classification (evidence)	Genetic testing method
1	6/F	Foot plantarflexion	Isolated	No	No	*GCH1*	c.317C>T	p.Thr106Ile	Missense variant	Het	NA	LP (PS1 + PM2 + PP3)	Targeted NGS
2	3/F	Foot inversion	Isolated	No	No	*GCH1*	c.608G>A	p.Gly203Glu	Missense variant	Het	NA	LP (PS1 + PM2 + PP3)	Targeted NGS
3	12/M	Foot inversion; foot plantarflexion	Isolated	No	No	*GCH1*	c.328C>T	p.Gln110Ter	Nonsense variant	Het	Maternal	P (PVS1 + PM2 + PP3)	Targeted NGS
4	23/F	Foot inversion; foot plantarflexion	Isolated	No	No	*GCH1*	c.327C>G	p.Tyr109Ter	Nonsense variant	Het	Maternal	P (PVS1 + PM2 + PP3)	Targeted NGS
5	5/F	Foot inversion	Isolated	No	No	*GCH1*	c.557C>A	p.Thr186Lys	Missense variant	Het	NA	LP (PS1 + PM2 + PP3)	WES
6	47/F	Foot inversion	Isolated	No	Yes	*GCH1*	c.631_632del	p.Met211ValfsTer38	Frameshift variant	Het	Maternal	P (PVS1 + PM2 + PP1 + PP3)	WES
7	24/F	Toe extension/striatal toe; foot inversion	Isolated	No	No	*PRKN*^a^	Deletion of exon 4	NA	CNV	Het	Paternal	P (PVS1 + PM2 + PM3)	MLPA
							Deletion of exon 3	NA	CNV	Het	Maternal	P (PVS1 + PM2 + PM3)	MLPA
8	26/M	Toe curling/flexion	Isolated	No	No	*PRKN*^a^	c.2T>C	p.Met1?	Missense variant	Het	Paternal	P (PVS1 + PM2 + PM3)	WES
							Deletion of exon 3	NA	CNV	Het	Maternal	P (PVS1 + PM2 + PM3)	MLPA
9	18/F	Toe curling/flexion	Isolated	No	No	*PRKN*	c.850G>C	p.Gly284Arg	Missense variant	Hom	NA	P (PS1 + PM2 + PM3 + PP3 + PP5)	WES
10	23/F	Leg extension	Combined	Parkinsonism	Yes	*PRKN*^a^	*c.668C>G*	p.Ser223Ter	Nonsense variant	Het	Paternal	P (PVS1 + PM2 + PM3 + PP3)	Targeted NGS
							Deletion of exon 5 and 6	NA	CNV	Het	Maternal	P (PVS1 + PM2 + PM3)	MLPA
11	19/F	Unclassed LLD	Combined	Parkinsonism	No	*PINK1*^a^	c.1474C>T	p.Arg492Ter	Nonsense variant	Het	Paternal	P (PVS1 + PM2 + PP3)	WES
							c.938C>T	p.Thr313Met	Missense variant	Het	Maternal	LP (PM2 + PM3 + PP3 + PP5)	WES
12	8/M	Foot inversion	Complex	Cognitive impairment; dysarthria	No	*PANK2*^a^	c.1502T>A	p.Ile501Asn	Missense variant	Het	Paternal	LP (PM1 + PM2 + PM3 + PP3)	WES
							c.833G>A	p.Arg278His	Missense variant	Het	Maternal	LP (PM1 + PM2 + PM3 + PP3)	WES

AAO, age at onset; F, female; M, male; het, heterozygous; CNV, copy-number variants; hom, homozygous; LLD, lower limb dystonia; ACMG, American College of Medical Genetics and Genomics; P, pathogenic; LP, likely pathogenic; NA, not available.

a Compound heterozygous variants.

**Table 3:** Overview of identified pathogenic and likely pathogenic variants in the LLD-plus group.

No.	AAO/sex	Initial LLD symptom	Dystonia type		Other associated disorders	Family history	Gene	cDNA	Protein	Variant type	Het/hom	Inherited	ACMG classification (evidence)	Genetic testing method
1	7/F	Foot plantarflexion	Generalized	Isolated	No	No	*TOR1A*	c.907_909del	p.Glu303del	In-frame indel	Het	NA	P (PS3 + PS4 + PM2 + PP3)	Targeted NGS
2	10/M	Foot plantarflexion	Segmental	Isolated	No	Yes	*TOR1A*	c.907_909del	p.Glu303del	In-frame indel	Het	NA	P (PS3 + PS4 + PM2 + PP3)	Targeted NGS
3	4/M	Leg external rotation	Generalized	Isolated	No	No	*TOR1A*	c.907_909del	p.Glu303del	In-frame indel	Het	NA	P (PS3 + PS4 + PM2 + PP3)	WES
4	6/F	Foot plantarflexion	Generalized	Isolated	No	No	*TOR1A*	c.907_909del	p.Glu303del	In-frame indel	Het	NA	P (PS3 + PS4 + PM2 + PP3)	WES
5	15/F	Foot plantarflexion	Generalized	Isolated	No	No	*TOR1A*	c.907_909del	p.Glu303del	In-frame indel	Het	NA	P (PS3 + PS4 + PM2 + PP3)	WES
6	16/F	Toe extension	Generalized	Isolated	No	No	*TOR1A*	c.907_909del	p.Glu303del	In-frame indel	Het	NA	P (PS3 + PS4 + PM2 + PP3)	WES
7	10/F	Foot Inversion	Multifocal	Isolated	No	No	*TOR1A*	c.907_909del	p.Glu303del	In-frame indel	Het	Maternal	P (PS3 + PS4 + PM2 + PP3)	WES
8	15/M	Foot plantarflexion	Generalized	Isolated	No	Yes	*TOR1A*	c.907_909del	p.Glu303del	In-frame indel	Het	NA	P (PS3 + PS4 + PM2 + PP3)	WES
9	7/F	Toe curling/flexion	Multifocal	Isolated	No	No	*KMT2B*	c.4458delC	p.Arg1487Alafs*7	Frameshift variant	Het	*De novo*	P (PVS1 + PS2 + PM2 + PP3)	WES
10	9/F	Unclassed bilateral LLD	Generalized	Isolated	No	No	*KMT2B*	c.7373-1G>A	NA	Splicing variant	Het	De novo	P (PVS1 + PS2 + PM2)	WES
11	6/F	Unclassed bilateral LLD	Generalized	Isolated	No	No	*KMT2B*	c.3140dupG	p.Ala1049Glyfs*39	Frameshift variant	Het	De novo	P (PVS1 + PS2 + PM2)	WES
12	2/M	Unclassed bilateral LLD	Generalized	Isolated	No	Yes	*KMT2B*	c.5462dupC	p.Leu1822Thrfs*12	Frameshift variant	Het	Paternal	LP (PVS1 + PM2)	WES
13	6/M	Unclassed bilateral LLD	Generalized	Isolated	No	Yes	*KMT2B*	c.5877_5878ins	p.Gly1962Trpfs*10	Frameshift variant	Het	Paternal	LP (PVS1 + PM2)	WES
14	3/M	Foot plantarflexion	Segmental	Isolated	No	No	*TH*	c.698G>A	p.Arg233His	Missense variant	Hom	NA	LP (PM1 + PM2 + PP3 + PP5)	WES
15	4/F	Unclassed bilateral LLD	Segmental	Complex	Cognitive impairment	Yes	*TH*^a^	c.457C>T	p.Arg153Ter	Nonsense variant	Het	Paternal	P (PVS1 + PM2 + PP3 + PP5)	WES
								c.67G>A	p.Ala23Thr	Missense variant	Het	Maternal	LP (PM1 + PM2 + PM3 + PP1)	WES
16	1/F	Leg extension	Generalized	Combined	Myoclonus	Yes	*SGCE*	c.304C>T	p.Arg102Ter	Nonsense variant	Het	NA	P (PVS1 + PM2 + PP3 + PP5)	WES
17	23/M	Foot inversion/plantarflexion	Segmental	Isolated	No	No	*PRKN*^a^	c.850G>C	p.Gly284Arg	Missense variant	Het	Paternal	P (PS1 + PM2 + PM3 + PP3 + PP5)	WES
								c.634T>C	p.Cys212Arg	Missense variant	Het	Maternal	LP (PM2 + PM3 + PM5 + PP3)	WES

AAO, age at onset; F, female; M, male; het, heterozygous; hom, homozygous; LLD, lower limb dystonia; ACMG, American College of Medical Genetics and Genomics; P, pathogenic; LP, likely pathogenic; NA, not available.

a Compound heterozygous variants.

The genetic architecture differed significantly between the LLD and LLD-plus groups. In the LLD group, *GCH1* was the most frequently implicated gene, accounting for 50.0% (6/12) of molecularly diagnosed cases, followed by *PRKN* (4/12, 33.3%). Other causative genes included *PINK1* (*n* = 1) and *PANK2* (*n* = 1). In contrast, *TOR1A* was the predominant causative gene in the LLD-plus group, found in 8 of 17 (47.1%) molecularly confirmed patients, followed by *KMT2B* (5/17, 29.4%). Additional causative genes in this group included *TH* (*n* = 2), *SGCE* (*n* = 1), and *PRKN* (*n* = 1). The proportion of patients harboring P/LP variants was higher in the LLD-plus group than in the LLD group (51.5% vs. 34.3%), although the difference did not reach statistical significance (*p* = 0.151).

Based on the genetic findings, patients with P/LP variants had a significantly younger age at onset than those without variants [median (IQR): 9.0 (5.5–18.5) vs. 15.5 (9.8–27.5) years; *p* = 0.012]. However, no significant differences were observed between the two groups in terms of positive family history, dystonia distribution, or the presence of associated features (Table 4).

**Table 4:** Comparison of clinical features between groups based on genetic findings.

Variable	Subgroup by genetic findings			*p*-value^*^
	Patients with P/LP	Patients with VUS	Patients without variants	
Total patients, *n* (%)	29 (42.6%)	5 (7.4%)	34 (50.0%)	
Sex, *n* (%)				0.089
Males	10 (34.5)	4 (80.0)	19 (55.9)	
Females	19 (65.5)	1 (20.0)	15 (44.1)	
AAO, y (median, IQR)	9.0 (5.5–18.5)	18.0 (2.0–31.5)	15.5 (9.8–27.5)	**0.012**
Positive family history, *n* (%)	8 (27.6)	0 (0.0)	4 (11.8)	0.111
AAO distribution, *n* (%)				**0.049**
Early-onset	23 (79.3)	4 (80.0)	19 (55.9)	
Adult-onset	6 (20.7)	1 (20.0)	15 (44.1)	
Dystonia distribution, *n* (%)				0.342
Focal	4 (13.8)	1 (20.0)	11 (32.4)	
Segmental	4 (13.8)	1 (20.0)	4 (11.8)	
Multifocal	10 (34.5)	3 (60.0)	11 (32.4)	
Generalized	11 (37.9)	0 (0.0)	8 (23.5)	
Associated features, *n* (%)				0.966
Isolated dystonia	24 (82.8)	3 (60.0)	28 (82.4)	
Combined/complex dystonia	5 (17.2)	2 (40.0)	6 (17.6)	
Symptom subgroup, *n* (%)				0.167
LLD group	12 (41.4)	3 (60.0)	20 (58.8)	
LLD-plus group	17 (58.6)	2 (40.0)	14 (41.2)	

*p-value represents the comparative analysis between the LP/P group and the group of patients without variants; cases with VUS (*n* = 5) were excluded from this comparative analysis to minimize uncertainty and avoid potential ambiguity. Significant difference was indicated in bold. AAO, age at onset; IQR, interquartile range; LP, likely pathogenic; P, pathogenic; VUS, variants of uncertain significance; LLD, lower limb dystonia.

### 3.3 Genotype–phenotype correlations

#### 3.3.1 TOR1A

In this cohort, eight patients were identified with *TOR1A* variants, all of which were the classic c.907_909delGAG variant. The variant was identified by targeted NGS in two patients and by WES in six patients, as detailed in Table 3. The majority of patients (5/8, 62.5%) exhibited dystonia onset in childhood, while the remaining three (3/8, 37.5%) presented in adolescence. The initial manifestations included foot plantarflexion (*n* = 5), foot inversion (*n* = 1), toe extension (*n* = 1), and leg external rotation (*n* = 1). Generalized dystonia developed in six patients, while the remaining two had multifocal and segmental dystonia, respectively. The spread of dystonia most commonly involved the trunk and/or upper limbs, while involvement of cranial or cervical muscles was uncommon. Six patients underwent deep brain stimulation (DBS), and all showed a favorable clinical response.

#### 3.3.2 GCH1

Six patients were identified as carrying pathogenic or likely pathogenic variants in the *GCH1* gene, including three missense variants (c.317C>T, c.608G>A, and c.557C>A), two nonsense variants (c.328C>T and c.327C>G), and one frameshift variant (c.631_632del). These variants were identified by targeted NGS in four patients and by WES in two patients, as detailed in Table 2. All patients presented with dystonia restricted to the lower limb(s). Disease onset occurred in childhood in four cases, whereas the remaining two patients developed symptoms in adulthood (at ages 23 and 47 years, respectively). The focal lower limb dystonic symptoms included foot plantarflexion (*n* = 1), foot inversion (*n* = 3), foot inversion plus foot plantarflexion (*n* = 2). All six patients showed an excellent and sustained response to low-dose levodopa, consistent with a diagnosis of dopa-responsive dystonia (DRD). Parkinsonism was not observed, except for postural tremor in one patient.

#### 3.3.3 KMT2B

We identified six patients harboring *KMT2B* variants, five of whom carried pathogenic or likely pathogenic variants, including four frameshift variants (c.4458delC, c.3140dupG, c.5462dupC, and c.5877_5878ins) and one splice-site variant (c.7373-1G>A). All five P/LP *KMT2B* variants were identified by WES. These variants have been previously reported by our group (14, 15). All patients developed dystonic symptoms in infancy or childhood and exhibited generalized or multifocal dystonia. Characteristic dysmorphic features were observed, including broad nasal bridge (*n* = 3), bulbous nasal tip (*n* = 2), short stature (*n* = 1), microcephaly (*n* = 1), and elongated face (*n* = 1). None of these patients exhibited global developmental delay or intellectual disability.

#### 3.3.4 PRKN

Six patients were identified with variants in *PRKN*, a gene typically associated with autosomal recessive early-onset Parkinson disease (EOPD). Among these patients, five carried biallelic pathogenic or likely pathogenic variants, and one carried compound heterozygous VUS variants. The molecular findings were heterogeneous and included CNVs, missense variants, and nonsense variants. The corresponding genetic testing methods for each molecular finding were provided in Tables 2, 3. Notably, the *PRKN* CNVs were detected by MLPA. Among the five patients with biallelic P/LP variants, one carried a CNV in a compound-heterozygous state, two carried missense variants, one carried a compound-heterozygous combination of a missense variant and a CNV, and one carried a compound-heterozygous combination of a nonsense variant and a CNV. The average age at onset was 22.8 years, ranging from 18 to 26 years. Four patients presented with dystonia limited to the lower limbs, whereas the fifth patient exhibited progressive dystonia involving the trunk. The initial dystonic symptoms included toe flexion (*n* = 2), leg extension (*n* = 1), foot inversion with plantarflexion (*n* = 1), and foot inversion with striatal toe (*n* = 1). Parkinsonism was observed in one of the five patients. This patient developed parkinsonism 2 years after the onset of lower limb dystonia and showed a relatively good response to levodopa therapy for approximately 5 years, followed by the development of dyskinesia.

## 4 Discussion

Lower limb dystonia (LLD) is a clinically significant subtype of dystonia that can substantially impair gait and cause cramping and aching pain. In this study, we characterized the clinical features and genetic profiles of LLD patients in mainland China. Consistent with previous studies, lower limb dystonia in our cohort showed a predominance of childhood or adolescent onset (4). Specially, 67.6% of patients developed symptoms before the age of 20 years. Among patients with early-onset, 63.0% experienced progression of dystonia from the lower limbs to other body regions, compared with only 18.2% among those with adult-onset. These findings support previous observations that early-onset LLD is associated with a high risk of generalization, whereas adult-onset LLD tends to remain focal (4). The clinical manifestations of LLD are heterogeneous and may include foot inversion or eversion, foot plantarflexion, toe flexion or extension, leg extension or flexion, and leg external or internal rotation (11). In our cohort, foot dystonia was the most common initial symptom, typically characterized by foot inversion and plantarflexion.

Genetic studies specifically focusing on LLD remain limited. Although several previous studies have investigated DYT1 mutations in part of patients with LLD (6, 11), comprehensive genetic characterization remains scarce. In this study, we systematically analyzed the genetic profiles of LLD patients using next-generation sequencing approach. Overall, 42.6% of the patients were identified with pathogenic or likely pathogenic variants, representing the molecular diagnostic yield observed in our cohort, while 7.4% were identified with VUS that need further investigation. Among patients with pathogenic or likely pathogenic variants, the majority (79.3%) exhibited early-onset disease. These findings indicate that genetic causes can be identified in a substantial proportion of patients with LLD in our cohort, particularly among patients with early-onset disease. However, given the heterogeneous genetic testing strategies used during the study period and the potential for ascertainment bias, the observed diagnostic yield should not be interpreted as an estimate of the prevalence or overall contribution of monogenic causes to LLD. Nevertheless, our findings support the potential value of genetic testing as an important diagnostic strategy, particularly in patients with early-onset LLD.

Our study identified *GCH1* as the most frequently identified genetic cause among genetically solved patients with focal lower limb dystonia in our cohort. Pathogenic variants in *GCH1* are a well-established genetic cause of dopa-responsive dystonia (DRD), a rare movement disorder characterized by an excellent and sustained response to low doses of levodopa (16). The clinical phenotype includes dystonia and parkinsonism, independently or combined. According to a MDSGene systematic review, 70% of heterozygous *GCH1* carriers present with isolated dystonia, 11% with pure parkinsonism, and 17% with combined dystonia-parkinsonism (17). Dystonia of feet or legs is the most frequent initial manifestation (17). In our cohort, all six patients with *GCH1* mutations presented with focal lower limbs dystonia and were reported to have an excellent response to low-dose levodopa therapy. However, because treatment dosage, duration, and therapeutic response were not systematically assessed using standardized measures in this retrospective study, these observations should be interpreted as retrospective qualitative clinical observations. *GCH1* variants accounted for 20.7% of all genetically solved cases and 50.0% of genetically solved patients in the LLD group in our cohort. Given the heterogeneous genetic testing strategies used during the study period, these proportions should be interpreted as cohort-specific frequencies rather than population-level prevalence estimates. Nevertheless, the consistent clinical response observed in our *GCH1*-positive patients highlights the potential treatability of *GCH1*-associated dystonia and supports consideration of DRD in children presenting with lower-limb dystonia, particularly when the clinical features are suggestive. A therapeutic levodopa trial may be considered in such patients, including while genetic testing results are pending.

Notably, variants in *PRKN* (commonly referred to as *Parkin*) and *PINK1* were also frequently identified in our cohort, especially in the LLD group. Mutations in *PRKN* and *PINK1* are probably the two most common causes of autosomal recessive early-onset Parkinson’s disease (10). They have very similar clinical presentations, encompassing parkinsonian features, dystonia, slow disease progression, and an excellent response to levodopa, often accompanied by early development of motor complications (18). In a large series of patients with *PRKN* mutations, dystonia was observed as the initial symptom in 41% of cases, predominantly affecting the lower limbs but uncommonly involving the neck, trunk, and hands (19). Antonio et al. further observed that isolated dystonia can last up to 16 years before the onset of parkinsonian symptoms in *Parkin* gene mutation carriers (9). In our cohort, five patients carried biallelic P/LP variants in *PRKN*, and one patient harbored compound heterozygous pathogenic variants in *PINK1*. Among them, one patient with *PRKN* variants and the patient with *PINK1* variants both developed parkinsonian symptoms within 3 years after the onset of dystonia. It is well established that dystonia may precede the typical motor symptoms of Parkinson’s disease by many years (up to 25 or longer), although in most cases PD will be diagnosed within 10 years after dystonia onset (10). Therefore, adult-onset isolated lower limb dystonia should warrant careful longitudinal clinical evaluation for the potential development of Parkinson’s disease.

In our cohort, *TOR1A* and *KMT2B* were identified as the most common genetic causes of the LLD-plus group. *TOR1A*-related dystonia and *KMT2B*-related dystonia are now recognized as two major forms of early-onset dystonia worldwide (7, 20). Lower limb dystonia is the most common initial manifestation, particularly in patients with *KMT2B* variants (7). The clinical picture of early onset and rapid generalization may serve as “red flags” that can prompt clinicians to prioritize genetic testing for *TOR1A* and *KMT2B*, thereby facilitating early diagnosis and appropriate management. Such management may include consideration of DBS, which has shown favorable therapeutic outcomes in these patients (21, 22).

This study has several limitations. First, the retrospective and non-consecutive recruitment design may have introduced recall and information biases, as well as potential selection bias. Second, the genetic testing strategies varied across the study period, including targeted NGS, WES, and MLPA. The different testing approaches have different genomic coverage and variant detection capabilities and may therefore have influenced the observed molecular diagnostic yield and the distribution of pathogenic/likely pathogenic variants across genes. Therefore, the reported diagnostic yield and gene frequencies should be interpreted as cohort-specific findings rather than population-level estimates. Third, the targeted NGS panel used in 2016 did not include several newly identified genes associated with dystonia, including *VPS16*, *KMT2B*, *EIF2AK2*, and *AOPEP*. This limitation may have contributed to the proportion of genetically unsolved cases. Furthermore, the sequencing approaches in this study may have failed to detect certain variant types, such as dynamic repeat expansions, deep intronic variants, and some structural variants or other complex genomic alterations. Finally, parental genetics testing, functional validation studies, and segregation analysis were not available for some patients, limiting the ability to confirm variant pathogenicity. Future studies incorporating comprehensive genomic approaches, including whole-genome sequencing and long-read sequencing, together with functional validation, are warranted to further elucidate the genetic architecture of LLD. In addition, treatment-response data were not systematically collected using standardized measures, including levodopa dosage and treatment duration or DBS target, follow-up duration, and standardized clinical rating scales. Therefore, treatment-response findings should be interpreted as retrospective qualitative observations.

## 5 Conclusion

In conclusion, this study expands the understanding of the clinical spectrum and genetic architecture of LLD. LLD is a clinically and genetically heterogeneous disorder, with a substantial proportion of patients harboring identifiable monogenic causes, particularly among those with early-onset disease. Incorporating genetic testing into the diagnostic workup may improve diagnostic accuracy and facilitate personalized management for patients with LLD.

## Data Availability

The raw data supporting the conclusions of this article will be made available by the authors, without undue reservation, to any qualified researcher.

## Footnotes

1
www.coffalyser.net
