# Supplementary material for: Clinical and genetic profile of lower limb dystonia: a dual-center retrospective study in China
Source: Front Neurol. 2026 Oct 30;17:1957431. doi: 10.3389/fneur.2026.1957431 (PMC13759380; doi:10.3389/fneur.2026.1957431)
Supplement: Supplementary file 1 [file Supplementary_file_1.DOCX]

**Supplementary Materials**

**Clinical and Genetic Profile of Lower Limb Dystonia: A Dual-Center Retrospective Study in China**

**Supplementary** Methods

**Whole exome sequencing (from January 2017 to December 2024)**

Genomic DNA was extracted from peripheral blood leukocytes and fragmented into 150-300 bp length fragments using an ultrasonoscope (Covaris S2, USA). The DNA fragments were then processed by end-repairing, A-tailing and adaptor ligation, PCR amplification, and enriched by the whole-exome panel. Paired-end sequencing was performed on llumina HiSeq X-ten platform to provide a mean sequence coverage of more than 100X and a coverage of at least 20X in more than 95% target areas. The generated data was then processed using bioinformatic tools. For the quality control, a few unqualified sequences from the primary data included 3′-/5′- adaptor sequences and low-quality reads were removed by the Cutadapt and FastQC, respectively. The remaining sequences were termed as clean reads for further analysis. The clean reads were then mapped to the reference human genome (UCSC hg19) using the BWA (Burrows Wheeler Aligner). Duplicated reads were marked and removed using Picard Tools by default parameters. Short read alignment and variants visualization were performed by the IGV (Integrative Genomics Viewer). SNVs and indels were identified using the GATK (The Genome Analysis Toolkit). Subsequent annotation was performed using ANNOVAR with various descriptive information, including function implication (gene region, functional effect, mRNA GenBank accession number, amino acid change, etc.) and allele frequency in population databases (1000 Genomes, ExAc and gnomAD). Only missense, nonsense, splice-site, in-frame insertion/deletion, and frameshift variants with population minor allele frequency (MAF) < 1% were selected for further assessment. Previously identified SNPs were determined using the NCBI dbSNP. Known disease-causing mutations were identified from the professional version of Human Gene Mutation Database (HGMD, http://www.hgmd.org) or from mutations previously reported in the literature. Unreported missense mutations were predicted by SIFT (http://sift.jcvi.org), PolyPhen-2 (http://genetics.bwh.harvard.edu/pph2), MutationTaster (http://www.mutationtaster.org) and CADD (http://cadd.gs.washington.edu). The phenotypes of the screened genes and the inherited modes were also taken into consideration in order to further exclude irrelevant genes. Then the variants left were verified by Sanger sequencing and tested in other family members. Interpretations of the variants were according to the American College of Medical Genetics and Genomics (ACMG) Standards and Guidelines. All the variants were categorized into pathogenic, likely pathogenic, uncertain significance, likely benign and benign.

**Targeted next-generation sequencing (from January 2016 to December 2016)**

The methods used for DNA target capture, enrichment and elution followed the manufacturer’s protocols. Briefly, genomic DNA was extracted from peripheral blood leukocytes, and shattered into fragments ranging from 150 bp to 300 bp using an ultrasonoscope (Covaris S2, USA). After end-repair, A-tailings and adaptors from Illumina were ligated to the ends. Successful adaptor ligation was confirmed by PCR in which primers contained a custom-synthesized barcode sequence as a sample index signature. The generating library was hybridized to the custom capture array (Agilent SureSelect), subsequently washed and eluted. The enriched DNA was then sent to high throughput sequencing conducted by Illumina Hiseq2500 platform according to the manufacture’s protocol. The generated data was then processed using bioinformatic tools. For the quality control, a few unqualified sequences from the primary data included 3′-/5′- adaptor sequences and low-quality reads were removed by the Cutadapt and FastQC, respectively. The remaining sequences were termed as clean reads for further analysis. The clean reads were then subjected to alignment against the reference human genome (GRCh37/hg19) using the BWA (Burrows Wheeler Aligner). The alignment files (BAM) were generated with SAM tools and the reads of low mapping quality (< Q30) were filtered out. Duplicated reads were marked and removed using Picard Tools by default parameters. Short read alignment and variants visualization were performed by the IGV (Integrative Genomics Viewer). SNVs and indels were identified using the GATK (The Genome Analysis Toolkit). Subquent annotation was performed using ANNOVAR with various descriptive information, including function implication (gene region, functional effect, mRNA GenBank accession number, amino acid change, etc.) and allele frequency in population databases (1000 Genomes, ExAc, and gnomAD). Previously identified SNPs were determined using the NCBI dbSNP. Known disease-causing mutations were identified from the professional version of Human Gene Mutation Database (HGMD, http://www.hgmd.org) or from mutations previously reported in the literature. Unreported missense mutations were predicted by (http://sift.jcvi.org), PolyPhen-2 (http://genetics.bwh.harvard.edu/pph2), MutationTaster (http://www.mutationtaster.org) and CADD (http://cadd.gs.washington.edu). The sequence variants were further interpreted and classified according to the American College of Medical Genetics and Genomics (ACMG) Standards and Guidelines. Potential causal variants (substitutions or indels) were verified by Sanger sequencing. All the variants were categorized into pathogenic, likely pathogenic, uncertain significance, likely benign and benign.

Table S1 Gene list of the panel of targeted next-generation sequencing (148 genes)

| ACTB | TAF1 | PANK2 | SLC6A3 |
| --- | --- | --- | --- |
| ANO3 | FA2H | PARK2 | SPR |
| ARX | FOXG1 | PLA2G6 | TH |
| ATP1A3 | GCH1 | PRKRA | THAP1 |
| ALDH5A1 | GNAL | SERAC1 | TOR1A |
| BCAP31 | MECP2 | SGCE | TUBB4A |
| CIZ1 | OPA3 | SLC30A10 | PRRT2 |
| ADCY5 | SLC2A1 | PNKD/MRI | VPS13A |
| ATP7B | FTL | C19orf12 | MTP |
| NPC1 | NPC2 | GLB1 | ATM |
| TIMM8A | SLC19A3 | WDR45 | ATN1 |
| HEXA | SPG11 | PINK1 | PARK7 |
| PLP1 | CP | CACNA1A | ATP13A2 |
| CBS | POLG | MTR | MTRR |
| ANG | DGKQ | IREB2 | HTRA2 |
| APP | DNAJC6 | LAMP2 | PSEN1 |
| ATP6AP2 | DNAJC13 | LRRK2 | PSEN2 |
| BDNF | DRD3 | LTF | RAB7L1 |
| BST1 | EIF4G1 | MAOA | SLC6A4 |
| CCK | FBXO7 | MAPT | SMPD1 |
| CCKAR | FMR1 | MTHFR | SNCA |
| CNR1 | FTH1 | NEFM | SV2C |
| COMT | GAK | NR4A2 | SYNJ1 |
| CRY1 | GBA | PARL | TEF |
| CYP27A1 | GBAP1 | PIK3CD | TFRC |
| DCTN1 | GDNF | GIGYF2 | TRPM2 |
| GRIN2A | GRN | PRKAG2 | UCHL1 |
| ADCK3 | DARS2 | KCNC3 | VPS35 |
| AFG3L2 | DNMT1 | KCND3 | SETX |
| ANO10 | EEF2 | NUBPL | SLC1A3 |
| ATCAY | FGF14 | PDYN | SPTBN2 |
| ATP2B3 | GBA2 | PEX2 | SRD5A3 |
| ATP8A2 | GJB1 | PLEKHG4 | SYNE1 |
| C10orf2 | GRID2 | PRKCG | SYT14 |
| CA8 | GRM1 | SACS | TDP1 |
| TTBK2 | ITPR1 | VLDLR | TGM6 |
| STUB1 | KCNA1 | ZNF592 | TPP1 |

Table S2 Overview of identified pathogenic and likely pathogenic variants in the LLD group

| No. | Gene | cDNA | Protein change | Het/Hom | Inherited | HGMD | Frequency GnomAD v2.1.1/exomes | Frequency 1000 Genomes | Frequency ExAC | SIFT score | Polyphen2 | MutationTaster | CADD v1.6 | ACMG Classification (evidence) |
| --- | --- | --- | --- | --- | --- | --- | --- | --- | --- | --- | --- | --- | --- | --- |
| 1 | *GCH1* | c.317C>T | p.Thr106Ile | Het | NA | Yes | - | - | - | 0 | PD | DC | 29.3 | LP (PS1+PM2+PP3) |
| 2 | *GCH1* | c.608G>A | p.Gly203Glu | Het | NA | Yes | - | - | - | 0.001 | PD | DC | 25.3 | LP (PS1+PM2+PP3)) |
| 3 | *GCH1* | c.328C>T | p.Gln110Ter | Het | Maternal | Yes | - | - | - | NA | NA | DC | 43 | P (PVS1+PM2+PP3) |
| 4 | *GCH1* | c.327C>G | p.Tyr109Ter | Het | Maternal | No | - | - | - | NA | NA | DC | 45 | P (PVS1+PM2+PP3) |
| 5 | *GCH1* | c.557C>A | p.Thr186Lys | Het | NA | Yes | - | - | - | 0 | PD | DC | 25.2 | LP (PS1+PM2+PP3) |
| 6 | *GCH1* | c.631_632del | p.Met211ValfsTer38 | Het | Maternal | Yes | - | - | - | NA | NA | DC | 33 | P (PVS1+PM2+PP1+PP3) |
| 7 | *PRKN** | deletion of exon 4 | NA | Het | Paternal | Yes | - | - | - | NA | NA | NA | NA | P (PVS1+PM2+PM3) |
|  |  | deletion of exon 3 | NA | Het | Maternal | Yes | - | - | - | NA | NA | NA | NA | P (PVS1+PM2+PM3) |
| 8 | *PRKN** | c.2T>C | p.Met1? | Het | Paternal | Yes | 0.00007762 | - | 0.0002745 | 0.002 | PD | DC | 27.1 | P (PVS1+PM2+PM3) |
|  |  | deletion of exon 3 | NA | Het | Maternal | Yes | - | - | - | NA | NA | NA | NA | P (PVS1+PM2+PM3) |
| 9 | *PRKN* | c.850G>C | p.Gly284Arg | Hom | NA | Yes | 0.00001195 | - | 0.000008454 | 0 | PD | DC | 28 | P (PS1+PM2+PM3+PP3+PP5) |
| 10 | *PRKN** | *c.668C>G* | p.Ser223Ter | Het | Paternal | Yes | - | - | - | NA | NA | DC | 36 | P (PVS1+PM2+PM3+PP3) |
|  |  | deletion of exon 5 and 6 | NA | Het | Maternal | Yes | - | - | - | NA | NA | NA | NA | P (PVS1+PM2+PM3) |
| 11 | *PINK1** | c.1474C>T | p.Arg492Ter | Het | Paternal | Yes | 0.00007985 | 0.00019968 | 0.00003320 | NA | NA | DC | 49 | P (PVS1+PM2+PP3) |
|  |  | c.938C>T | p.Thr313Met | Het | Maternal | Yes | 0.00001990 | 0.00019968 | 0.000008293 | 0.014 | PD | DC | 28.2 | LP (PM2+PM3+PP3+PP5) |
| 12 | *PANK2** | c.1502T>A | p.Ile501Asn | Het | Paternal | Yes | - | - | - | 0 | PD | DC | 26.7 | LP (PM1+PM2+PM3+PP3) |
|  |  | c.833G>A | p.Arg278His | Het | Maternal | Yes | 0.000003977 | - | - | 0.025 | PD | DC | 29.3 | LP (PM1+PM2+PM3+PP3) |

CADD, Combined Annotation Dependent Depletion (https://cadd.gs.washington.edu/); GnomAD (https://gnomad.broadinstitute.org, version4.1.0); SIFT (http://sift.jcvi.org); PolyPhen-2 (http://genetics.bwh.harvard.edu/pph2); MutationTaster (http://www.mutationtaster.org); HGMD (https://www.hgmd.cf.ac.uk). * Compound heterozygous variants.

Abbreviations: het, heterozygous; hom, homozygous; ACMG, American College of Medical Genetics and Genomics; P, pathogenic; LP, likely pathogenic; PD, probably damaging; DC, disease causing; NA, not available.

Table S3 Overview of identified pathogenic and likely pathogenic variants in the LLD-plus group

| No. | Gene | cDNA | Protein | Het/Hom | De novo/inherited | HGMD | Frequency GnomAD v2.1.1/exomes | Frequency 1000 Genomes | Frequency ExAC | SIFT score | Polyphen2 | MutationTaster | CADD v1.6 | ACMG classification (evidence) |
| --- | --- | --- | --- | --- | --- | --- | --- | --- | --- | --- | --- | --- | --- | --- |
| 1 | *TOR1A* | c.907_909del | p.Glu303del | Het | NA | Yes | 0.0001153 | - | 0.00008237 | NA | NA | DC | 22.3 | P (PS3+PS4+PM2+PP3) |
| 2 | *TOR1A* | c.907_909del | p.Glu303del | Het | NA | Yes | 0.0001153 | - | 0.00008237 | NA | NA | DC | 22.3 | P (PS3+PS4+PM2+PP3) |
| 3 | *TOR1A* | c.907_909del | p.Glu303del | Het | NA | Yes | 0.0001153 | - | 0.00008237 | NA | NA | DC | 22.3 | P (PS3+PS4+PM2+PP3) |
| 4 | *TOR1A* | c.907_909del | p.Glu303del | Het | NA | Yes | 0.0001153 | - | 0.00008237 | NA | NA | DC | 22.3 | P (PS3+PS4+PM2+PP3) |
| 5 | *TOR1A* | c.907_909del | p.Glu303del | Het | NA | No | 0.0001153 | - | 0.00008237 | NA | NA | DC | 22.3 | P (PS3+PS4+PM2+PP3) |
| 6 | *TOR1A* | c.907_909del | p.Glu303del | Het | NA | Yes | 0.0001153 | - | 0.00008237 | NA | NA | DC | 22.3 | P (PS3+PS4+PM2+PP3) |
| 7 | *TOR1A* | c.907_909del | p.Glu303del | Het | Maternal | Yes | 0.0001153 | - | 0.00008237 | NA | NA | DC | 22.3 | P (PS3+PS4+PM2+PP3) |
| 8 | *TOR1A* | c.907_909del | p.Glu303del | Het | NA | Yes | 0.0001153 | - | 0.00008237 | NA | NA | DC | 22.3 | P (PS3+PS4+PM2+PP3) |
| 9 | *KMT2B* | c.4458delC | p.Arg1487Alafs*7 | Het | De novo | No | - | - | - | NA | NA | DC | 29.1 | P (PVS+PS2+PM2+PP3) |
| 10 | *KMT2B* | c.7373-1G>A | NA | Het | De novo | No | - | - | - | NA | NA | NA | 32 | P (PVS1+PS2+PM2) |
| 11 | *KMT2B* | c.3140dupG | p.Ala1049Glyfs*39 | Het | De novo | No | - | - | - | NA | NA | DC | 33 | P (PVS1+PS2+PM2) |
| 12 | *KMT2B* | c.5462dupC | p.Leu1822Thrfs*12 | Het | Paternal | No | - | - | - | NA | NA | DC | 23.2 | LP (PVS1+PM2) |
| 13 | *KMT2B* | c.5877_5878insC | p.Gly1962Trpfs*10 | Het | Paternal | No | - | - | - | NA | NA | DC | 33 | LP (PVS1+PM2) |
| 14 | *TH* | c.698G>A | p.Arg233His | Hom | NA | Yes | 0.0001063 | - | 0.0001230 | 0 | PD | DC | 27.4 | LP (PM1+PM2+PP3+PP5) |
| 15 | *TH** | TH c.457C>T | p.Arg153Ter | Het | Paternal | Yes | 0.00006217 | - | 0.00002810 | NA | NA | DC | 32 | P (PVS1+PM2+PP3+PP5) |
|  |  | c.67G>A | p.Ala23Thr | Het | Maternal | No | 0.0002208 | - | 0.0002346 | 0.165 | Benign | Polymorphism | 19.76 | LP (PM1+PM2+PM3+PP1) |
| 16 | *SGCE* | c.304C>T | p.Arg102Ter | Het | NA | Yes | - | - | - | NA | NA | DC | 38 | P (PVS1+PM2+PP3+PP5) |
| 17 | *PRKN** | c.850G>C | p.Gly284Arg | Het | Paternal | Yes | 0.00001195 | - | 0.000008454 | 0 | PD | DC | 28 | P (PS1+PM2+PM3+PP3+PP5) |
|  |  | c.634T>C | p.Cys212Arg | Het | Maternal | Yes | - | - | - | 0 | PD | DC | 27.8 | LP (PM2+PM3+PM5+PP3) |

CADD, Combined Annotation Dependent Depletion (https://cadd.gs.washington.edu/); GnomAD (https://gnomad.broadinstitute.org, version2.1.1); SIFT (http://sift.jcvi.org); PolyPhen-2 (http://genetics.bwh.harvard.edu/pph2); MutationTaster (http://www.mutationtaster.org); HGMD (https://www.hgmd.cf.ac.uk). * Compound heterozygous variants.

Abbreviations: het, heterozygous; hom, homozygous; ACMG, American College of Medical Genetics and Genomics; P, pathogenic; LP, likely pathogenic; PD, probably damaging; DC, disease causing; NA, not available.

Table S4 Overview of identified variants of uncertain significance (VUS) in the LLD group

| No. | AAO/sex | Dystonia type | Other associated disorders | Family History | Gene | cDNA | Protein | Het/Hom | De novo/inherited | HGMD | Frequency GnomAD v2.1.1/exomes | Frequency 1000 Genomes | Frequency ExAC | SIFT score | Polyphen2 | MutationTaster | CADD v1.6 |
| --- | --- | --- | --- | --- | --- | --- | --- | --- | --- | --- | --- | --- | --- | --- | --- | --- | --- |
| 1 | 3/M | Isolated | No | No | *ANO3* | c.101G>A | p.Arg34Gln | Het | Maternal | No | 0.000003981 | - | - | 0.009 | PD | DC | 28.5 |
| 2 | 20/M | Isolated | No | No | *PLA2G6** | c.1813C>A | p.Pro605Thr | Het | Paternal | No | - | - | - | 0.098 | PD | DC | 24.1 |
|  |  |  |  |  |  | c.967G>A | p.Val323Met | Het | Maternal | No | 0.000005844 | - | - | 0.002 | PD | DC | 25.4 |
| 3 | 43/F | Isolated | No | No | *PLA2G6** | c.1511C>T | p.Ser504Leu | Het | NA | No | - | - | - | 0.358 | Benign | DC | 23.4 |
|  |  |  |  |  |  | c.2065A>T | p.Ile689Phe | Het | NA | Yes | - | - | - | 0.015 | Benign | DC | 20.3 |

CADD, Combined Annotation Dependent Depletion (https://cadd.gs.washington.edu/); GnomAD (https://gnomad.broadinstitute.org, version4.1.0); SIFT (http://sift.jcvi.org); PolyPhen-2 (http://genetics.bwh.harvard.edu/pph2); MutationTaster (http://www.mutationtaster.org); HGMD (https://www.hgmd.cf.ac.uk). * Compound heterozygous variants.

Abbreviations: AAO, age at onset; F, female; M, male; het, heterozygous; hom, homozygous; ACMG, American College of Medical Genetics and Genomics; VUS, variant of uncertain significance; PD, probably damaging; DC, disease causing.

Table S5 Overview of identified variants of uncertain significance (VUS) in the LLD-plus group

| No. | AAO/sex | Dystonia type | Other associated disorders | Family History | Gene | cDNA | Protein | Het/Hom | De novo/inherited | HGMD | Frequency GnomAD v2.1.1/exomes | Frequency 1000 Genomes | Frequency ExAC | SIFT score | Polyphen2 | MutationTaster | CADD v1.6 |
| --- | --- | --- | --- | --- | --- | --- | --- | --- | --- | --- | --- | --- | --- | --- | --- | --- | --- |
| 1 | 1/M | Isolated | No | No | *KMT2B* | c.217C>T | p.Leu73Phe | Het | Maternal | No | - | - | - | 0 | Benign | Polymorphism | 20 |
| 2 | 18/M | Combined | Parkinsonism | No | *PRKN** | c.850G>C | p.Gly284Arg | Het | NA | Yes | 0.00001195 | - | 0.000008454 | 0 | PD | DC | 28 |
|  |  |  |  |  |  | c.507C>G | p.Cys169Trp | Het | NA | No | - | - | - | 0.001 | PD | DC | 25.9 |

CADD, Combined Annotation Dependent Depletion (https://cadd.gs.washington.edu/); GnomAD (https://gnomad.broadinstitute.org, version4.1.0); SIFT (http://sift.jcvi.org); PolyPhen-2 (http://genetics.bwh.harvard.edu/pph2); MutationTaster (http://www.mutationtaster.org); HGMD (https://www.hgmd.cf.ac.uk). * Compound heterozygous variants.

Abbreviations: AAO, age at onset; F, female; M, male; het, heterozygous; hom, homozygous; ACMG, American College of Medical Genetics and Genomics; VUS, variant of uncertain significance; PD, probably damaging; DC, disease causing; NA, not available.
